# The use of venous-specific preference based measures in health economic evaluation: Comparing apples and pears?

**DOI:** 10.1177/02683555211036250

**Published:** 2021-08-09

**Authors:** Luke Geoghegan, Sarah Onida, Alun H Davies

**Affiliations:** Section of Vascular Surgery, Department of Surgery and Cancer, Imperial College London, Imperial College London, London, UK

There has been a growing interest in the use of condition-specific preference-based measures (CSPBMs) in health economic assessment over the past two decades.^1^ CSPBMs provide a classification of a patient’s self-reported health state using disease-specific items and a value set that enables the utility of the described health state to be determined. The utility, or value, of a health state represents its desirability and is conventionally scored on a scale from 0 (equivalent to death) to 1 (equivalent to full health). Utilities comprise the quality component of quality-adjusted life years (QALYS) and are central in cost-effectiveness analyses of interventions. In the context of CSPBMs, utility values are derived by asking members of a population to rank response options for a selected patient reported outcome measure (PROM), such as the Aberdeen Varicose Vein Questionnaire (AVVQ) by their desirability, e.g. living with slight ankle swelling or living with pain for most days over the past two weeks. Using techniques such as time trade off and standard gamble, the preferences for certain health states described in the AVVQ (or any condition specific PROM) can be mapped onto a 0-1 scale.

A conventional method for determining utility values for QALYs is through generic preference-based measures such as the EuroQol five dimension (EQ-5D).^2^ The National Institute for Health and Care Excellence (NICE) expresses preference for the use of the EQ-5D-3L in its Technology Appraisal programme.^3^ Generic preference-based measures are designed to be relevant to all patient groups as they cover core components of health. The underlying assumption is that diseases and interventions produce measurable change in the domains of health covered by the generic health state classification system. This assumption may not hold for all conditions where generic PBMs are not sensitive to diseases-specific improvements in health. For example, the EQ-5D may not be sensitive to detect change in ankle swelling, itch and skin discolouration following intervention for venous disease, meaning the effectiveness of interventions may be undervalued when measured using the EQ-5D. Indeed, there is evidence to suggest that the responsiveness of the EQ-5D is poor in patients with venous leg ulcers.^4^ In contrast, the Aberdeen Varicose vein Questionnaire (AVVQ), a condition specific measure, has been shown to have good internal consistency, construct validity (when compared to the Varicose Vein Severity Score) and responsiveness.^5^ Further, the use of the EQ-5D-3L may not be appropriate for mild conditions due to an established ceiling effect where the instrument is not able to fully discriminate between health statuses of generally well individuals.^6^

The selection of the most sensitive tool to assess the effect of an intervention seems intuitive. Indeed, previous work has demonstrated that the use of CSPBMs in multiple sclerosis and cancer is more sensitive to detect mild impairment compared to the EQ-5D.^7^ However, the superior sensitivity of CSPBMs to specific diseases comes at the cost of limited comparability of utility values across conditions. Despite being measured on the same 0-to-1 scale, utility values derived using a CSPBM may not be directly comparable to utility values derived from generic measures. The use of disease-related items rather than generic items narrows the scope of valuation. The presence of a venous ulcer may seem less important when presented in a wider context of health considering factors such as mobility and mental wellbeing. However, when the wider context of health is not considered, the presence of a venous ulcer may be perceived as more problematic, leading to a downward bias for utilities viewed using CSPBMs.^8^ This is analogous to perspective distortion, a concept that describes how an object can appear markedly different with changes in camera angle and focal length. Further, a CSPBM may be insensitive in capturing the effect of co-morbidities or the side effects of interventions. This may result in an upward bias on utility values quantified using CSBPMs.^8^ The potential for differences in utility values arising from the use of generic and condition-specific PBMs may limit comparison of intervention cost-effectiveness across diseases, which has obvious implications for resource allocation.

The use of CSPBMs represents a tradeoff between having a measure that is more sensitive to aspects of health that are relevant to the population of interest and potential loss of comparability of utility values. There is some evidence to suggest that condition specific measures are better able to respond to changes experienced by patients with venous ulcers over time, compared to generic measures.^4^ However, it is not known whether this difference has any implications for resource allocation. Future work should determine whether utility values derived using CSPBMs differ from utility values derived using generic measures in venous disease. If generic measures are shown to miss important differences in the valuation of health states experienced by patients with venous disease, the use of CSPBMs is justified. CSPBMs can be used retrospectively to determine QALYs for specific cohorts in which the venous-specific measure has been used and prospectively in future clinical studies. This may yield more accurate estimates of QALYs gained or lost following venous intervention.

In summary, generic measures of health were not designed to provide a complete picture of health-related quality of life. One must consider whether it possible for a single instrument to capture everything that matters to all individuals. Further work is required to assess the psychometric properties of generic and condition specific measures across the spectrum of venous disease. The potential limited comparability of utility values derived using CSPBMs should not be seen as prohibitive, and the role for CSPBMs in cost-effectiveness analyses of venous interventions should be determined. The use of CSPBMs may lead to a better understanding of the value of interventions for venous disease, which may improve resource allocation in budget constrained healthcare systems.
